# Effects of dietary paddy rice on growth performance, carcass traits, bare skin color, and nutrient digestibility in geese

**DOI:** 10.1016/j.psj.2022.101865

**Published:** 2022-03-23

**Authors:** J. Yu, H. Zhang, H.M. Yang, Z.Y. Wang

**Affiliations:** ⁎ College of Animal Science and Technology, Yangzhou University, Yangzhou, Jiangsu Province 225009, P. R. China; † Joint International Research Laboratory of Agriculture and Agri-Product Safety of Ministry of Education of China, Yangzhou University, Yangzhou, Jiangsu Province 225000, P. R. China

**Keywords:** paddy rice, goose, growth performance, meat quality, bare skin color

## Abstract

The aim of this study was to investigate the effects of dietary paddy rice on growth performance, carcass traits, bare skin color, and nutrient digestibility in geese. A total of 300 twenty-eight-day-old male goslings were randomly divided into 5 treatment groups with 6 pens containing 10 goslings each. The geese were raised for 42 d on feed with 0% (control), 13, 26, 39, or 52% dietary paddy rice inclusion. Body weight and feed intake per pen were recorded from the arrival of goslings to the end of the trial. On d 70, two goslings were selected from each pen, one of which was used to measure slaughter performance, meat quality, meat proximate composition, and bare skin color, and one was used to determine nutrient utilization. The results showed that goslings fed a diet containing 26, 39, and 52% paddy rice had a higher final body weight, average daily feed intake, and average daily gain than those in the control group (*P* < 0.05). The abdominal fat yield in dietary paddy rice groups was higher than that of the control group (*P* < 0.05). There were no effects on the moisture, protein, and fat contents of breast and thigh muscle among the five treatments (*P* > 0.05). Compared to the control group, the breast muscle of geese fed the paddy rice had a lower L* value and a higher a* value (*P* < 0.05). Dietary paddy rice decreased the bill score of geese (*P* < 0.05). Geese in the paddy rice groups exhibited higher total starch digestibility than geese in the control group (*P* < 0.05). The digestibility of energy, crude protein, crude fat, crude fiber, NDF, and ADF did not differ between groups (*P* > 0.05). In conclusion, paddy rice is an excellent energy source in geese diets and could improve the growth performance, breast muscle color, and utilization of total starch but increase the abdominal fat yield and decrease the bill color of geese.

## INTRODUCTION

Corn is the main energy source in the poultry diet, accounting for approximately 60% of feed ingredients (Donohue and Cunningham, 2009). Currently, the global demand for corn is increasing at a rapid rate, not only for agricultural feed but also for fuel (Edgerton, 2009). The imbalance between the supply and demand of corn is causing its price to rise. Consequently, some poultry producers have sought local alternative raw materials that could replace corn in poultry diets without adversely affecting performance.

Paddy rice is one of the three major crops in China. According to statistics, 210 million tons of paddy rice was produced in China in 2019, which accounted for 27.98% of the global rice production (FAO, 2019). Among them, early *indica* rice is widely planted in southern China, where it is particularly predominant as an early-season crop (Song et al., 2006). Early *indica* rice is poor in taste and low in price because of its short growing period and is generally used as an industrial grain or reserve grain. Thus, it can be used as a good energy source to replace corn in poultry feed.

Several studies on broilers have shown that paddy rice is a good energy feed and can partially replace corn in the diet (Nanto et al., 2012; Sittiya and Yamauchi, 2014; Sittiya et al., 2014, 2015). Although paddy rice and corn are similar in energy and crude protein content, they differ in their starch and protein composition. Rice contains higher amylopectin and smaller starch granule sizes than corn, making it more easily digested by animals than corn (Tester et al., 2004; Svihus et al., 2005). The predominant protein in corn is prolamin, while that in rice is glutelin (Li, 2013). Glutelin is easier to digest and exhibits better antioxidant capacity than prolamin (Wang et al., 2016). However, the use of paddy rice can cause severe problems because of its high crude fiber content (approximately 10%) and nonstarch polysaccharides (**NSPs**, such as arabinoxylan and β-glucan) (Wang et al., 2008). Geese have an advantage over chickens in the use of crude fiber feed due to their strong gizzard. As herbivorous poultry, geese depend on dietary fiber for normal performance (Li et al., 2017). The unique digestive ability of geese can weaken the adverse effects of paddy rice crude fiber. In addition, paddy rice may affect goose skin color due to a lack of xanthophylls and carotenoids. Skin color is a major concern issue for consumers in the marketplace, especially in China (Chen et al., 2020).

Therefore, the objective of this study was to investigate the effect of replacing corn with different concentrations of paddy rice on growth performance, carcass traits, bare skin color, and nutrient digestibility in geese.

## MATERIALS AND METHODS

### Ethics Statement

All animal care and experimental procedures in this study were performed in accordance with the Regulations for the Administration of Affairs Concerning Experimental Animals of the People's Republic of China and approved by the animal care and use committee of Yangzhou University (Yangzhou, China).

### Experimental Diets and Design

Early *indica* rice, the first season paddy rice on the market, was selected as the experimental raw material. The paddy rice harvested in July 2019 was obtained from Shangrao City (Jiangxi Province, China). Whole grains were crushed and formulated into the diet. The chemical compositions of the paddy rice and corn are shown in Table 1.

**Table 1:** Analyzed nutrient content of paddy rice and corn in this study (%).

Items	Paddy rice	Corn
Gross energy (MJ/kg)	15.88	14.78
bulk density (g/L)	589.50	688.70
Dry matter /%	88.45	88.30
Crude protein	8.19	8.52
Crude fat	2.12	3.43
Ash	3.40	1.68
Crude fiber	11.25	2.49
NDF	26.46	12.59
ADF	14.21	3.47
Calcium	0.06	0.03
Total phosphorus	0.25	0.27
Total starch	61.79	55.52

This experiment was conducted at Yangzhou University Experimental Farm (Gaoyou, China) from October to December 2019. Three-hundred 28-day-old healthy male Jiangnan White goslings were obtained from a commercial hatchery (Suqian Lihua Animal Husbandry Co. Ltd., Shuyang, China). The goslings were all of similar body weights and were randomized to 5 groups that included 6 replicates per treatment and ten goslings per replicate. The geese were raised for 42 d on feed with 0% (control), 13, 26, 39, or 52% dietary early *indica* rice inclusion. These experimental diets (mash form) were formulated to be isonitrogenous and isocaloric and meet or exceed the nutrient requirements of geese according to the NRC (1994) and our prior research results (Shi et al., 2007; Wang et al., 2010). The composition and nutrient levels of the experimental diets are listed in Table 2. The geese were exposed to natural daylight, and the room temperature was maintained at approximately 24 ± 2°C. Water and feed were provided ad libitum throughout the experiment.

**Table 2:** Composition and nutrient levels of the experimental diets for d 28 to 70 (as-fed basis).

	Dietary paddy rice				
Items	0	13	26	39	52
Ingredient, %					
Corn	57.25	47.80	38.40	28.90	19.52
Soybean meal	23.81	24.09	24.40	24.68	24.97
Paddy rice	0.00	13.00	26.00	39.00	52.00
Rice husk	5.63	4.21	2.80	1.39	0.00
Wheat bran	9.81	7.40	4.90	2.52	0.00
Limestone	1.28	1.28	1.29	1.30	1.31
Calcium hydrogen phosphate	0.76	0.77	0.78	0.79	0.80
DL-Methionine	0.12	0.12	0.11	0.11	0.10
L-lysine.HCl	0.04	0.03	0.02	0.01	0.00
Salt	0.30	0.30	0.30	0.30	0.30
Premix^1^	1.00	1.00	1.00	1.00	1.00
Nutrient level^2^, %					
Metabolizable Energy (MJ/kg)	10.83	10.83	10.83	10.83	10.83
Crude protein	16.52	16.68	16.48	16.67	16.34
Crude fiber	5.77	5.96	5.87	5.80	5.80
Calcium	0.74	0.77	0.74	0.76	0.74
Total phosphorus	0.59	0.58	0.57	0.57	0.56
Lysine	0.87	0.87	0.87	0.87	0.87
Methionine	0.38	0.38	0.38	0.38	0.38
Total starch	32.67	32.86	34.16	35.68	35.86

1 Provided per kilogram of complete diet: 12,000 IU vitamin A (retinol), 4,000 IU vitamin D (rachitasterol), 18 IU vitamin E (D-a-tocopherol), 1.5 mg vitamin K (coagulation vitamin), 0.6 mg vitamin B_1_ (thiamine), 6 mg vitamin B_2_ (riboflavin), 2 mg vitamin B_6_ (pyridoxine), 0.01 mg vitamin B_12_ (cobalamin), 30 mg nicotinic acid, 9 mg *D*-pantothenic acid, 0.04 mg biotin, 0.5 mg folic acid, 60 mg Fe (ferrous sulfate), 10 mg Cu (copper sulfate), 95 mg Mn (manganese sulfate), 90 mg Zn (zinc sulfate), 0.5 mg I (potassium iodide), and 0.3 mg Se (sodium selenite), 350 mg choline.

2 Analyzed values except for metabolizable energy, lysine, and methionine.

### Sample Collection and Preparations

The feed intake was measured in replicates on a daily basis, and the BW of goslings was recorded at 28 and 70 d of age. The average daily feed intake (**ADFI**), average daily gain (**ADG**), and feed-to-gain ratio (**F/G**) were calculated at the end of the experiment. At d 70, one goose in each replicate with the average BW of the replicate was selected and exsanguinated after fasting for 6 h. After bleeding and plucking, the weight was recorded as the carcass weight. The geese were then eviscerated, and the semi-eviscerated carcass, eviscerated carcass, breast muscle (pectoralis major and minor), leg muscle (thigh and drumstick), and abdominal fat (fat around the abdomen and gizzard) were weighed. The semi-eviscerated weight was calculated by removing the trachea, esophagus, intestines, spleen, pancreas, gallbladder, reproductive organs, and gizzard contents and corneum from the carcass. The eviscerated weight was calculated by removing the heart, liver, proventriculus, gizzard, lungs, and abdominal fat from the semi-eviscerated carcass. The carcass, semi-eviscerated carcass, eviscerated carcass, breast muscle, thigh muscle, and abdominal fat percentages were calculated relative to the live BW before slaughter. After weighing, the major part of left breast and leg muscles (pectoralis major and thigh) were immediately divided into 2 pieces along the direction of muscle fibers. One part was stored at 4°C for measuring meat quality, and the other part was frozen at −20°C until analysis of the meat proximate composition. Additionally, the yellowness of the goose bare skin, including the flipper and bill, was scored.

The total excreta collection method was used to determine the feed nutrient utilization of geese (Matterson, 1965). At 70 d of age, one goose from each pen (6 geese per treatment) with the average BW of the pen was selected and housed separately in wire floor metabolism cages. Each cage was equipped with an individual feeder, a nipple drinker, and a plate underneath the cage to collect the excrement. The housing temperature was maintained at 24 ± 2°C, and geese were allowed ad libitum access to water and the experimental diets. The whole experiment involved a 5-d adaptation and 4-d collection period. The collected excreta were subjected to nitrogen fixation by adding l0 mL of 10% hydrochloric acid per 100 g of excreta. The excreta samples were then dried to a constant weight in an oven at 65°C, rewetted in atmospheric moisture for 24 h, weighed and ground to pass through a 40-mesh sieve. The experimental diets and the excrement were frozen and stored at −20°C for laboratory analysis.

### Meat Euality

The meat color was measured at 45 min postmortem with a chroma meter (Konica Minolta, CR-400, Osaka, Japan) and reported according to the Commission Internationale de l'Eclairage (**CIE**) system values of lightness (L*), redness (a*), and yellowness (b*). The pH value was recorded 45 min after slaughter using a portable pH meter (pH-STAR, Matthaus, Berlin, Germany). According to the method of Xiao et al. (2021), the cooking loss and shear force were measured using a thermostatic water bath (DK-S24, Shanghai Jinghong Experimental Equipment Co. Ltd, Shanghai, China) and a digital tenderness meter (C-LM3B, Tenovo, Beijing, China). Each index of each sample was measured repeatedly 3 times, and its average value was taken for statistical analysis.

### Bare Skin Color Score

The bare skin colors of geese corresponded to scores of 1 to 6 on the egg yellowness chart (Robotmation Co. Ltd., Tokyo, Japan), so this was chosen as the scoring standard. The higher the color score is, the more yellow the bare skin color; the lower the score is, the paler the color. Then, the goose flippers and bills were scored by comparison with the color chart.

### Chemical Analysis

The chemical composition of meat samples was analyzed according to the procedures set forth by the Association of Official Analytical Chemists (AOAC, 2000). Crude moisture was determined by oven drying at 100°C. Crude protein (N × 6.25) was measured by the Kjeldahl method with the Kjeltec System 8400 (FOSS NIRSystems Inc., Hillerød, Denmark). Crude fat (method 920.39) was measured by extraction with anhydrous ether.

All chemical analyses (diets, excrements) were performed in duplicate. Gross energy was determined using a Parr 6100 oxygen bomb calorimeter (Parr Instrument Company, Moline, IL) standardized with benzoic acid. The crude protein and crude fat contents in the diets and excrement samples were determined as described in the muscle above. The contents of crude fiber of the samples were determined using AOAC (2000) procedures. Neutral detergent fiber (**NDF**) and acid detergent fiber (**ADF**) were determined sequentially as described by Van Soest et al. (1991). The total starch content of the samples was measured using a commercial kit (Nanjing Jiancheng Bioengineering Institute, Nanjing, China) according to the manufacturer's instructions. The utilization of these nutrients was calculated using the following formula: Nutrient utilization = (Feed intake ×  Nutrient_diet_ − Excreta output × Nutrient_excrement_)/(Feed intake × Nutrient_excrement_) × 100%.

### Statistical Analysis

SPSS version 22.0 (SPSS, Inc., Chicago, IL) was used for data analysis. All data were tested for normal distribution by the Kolmogorov-Smirnov test. The flipper score data were not normally distributed, and they were analyzed by the nonparametric Kruskal-Wallis test. The data of other indicators were normally distributed, and they were subjected to one-way ANOVA. The significant differences between treatments were detected by Duncan's multiple range tests. Each replicate pen served as an experimental unit for all statistical analyses. When Kruskal-Wallis tests were used, the results are expressed as medians (interquartile range) and presented in figures with box plots (median, first, and third quartiles); other data are presented as the mean value and standard error of the means (**SEM**). Differences were considered statistically significant at *P* < 0.05.

## RESULTS

### Growth Performance

The BW, ADFI, ADG, and F/G of geese fed graded concentrations of dietary paddy rice are shown in Table 3. The goslings fed a diet containing 26, 39, and 52% paddy rice had a higher final BW, ADFI, and ADG than those in the control group (*P* < 0.05). However, increasing dietary paddy rice did not affect the F/G during the whole experimental period (*P* > 0.05).

**Table 3:** Effects of paddy rice on body weight (BW), average daily feed intake (ADFI), average daily gain (ADG), and feed/gain ratio (F/G) of goslings from 28 to 70 d of age^1^.

	Dietary paddy rice						
Item	0	13	26	39	52	SEM	*P-*value
Live weight, g							
D 28	1,649	1,648	1,648	1,648	1,647	0.771	0.993
D 70	3,847^a^	3,912^ab^	4,028^cd^	3,982^bc^	4,102^d^	22.16	<0.001
ADFI, g/d/bird	228.9^a^	236.6^ab^	242.7^b^	240.5^b^	244.1^b^	2.596	0.005
ADG, g/d/bird	52.34^a^	53.90^ab^	56.67^cd^	55.60^bc^	58.44^d^	0.784	<0.001
F:G, g/g	4.37	4.40	4.29	4.33	4.18	0.058	0.120

a–d Means with different superscripts within the same row indicate statistically significant difference (*P* < 0.05).

1 Each value represents the mean of 6 replicate pens.

### Slaughter Performance and Proximate Composition

As shown in Table 4, abdominal fat yield in dietary paddy rice groups was higher than that of the control group (*P* < 0.05). There were no effects on carcass yield, semi-eviscerated yield, eviscerated yield, breast muscle yield, or leg muscle yield (*P* > 0.05).

**Table 4:** Effects of paddy rice on slaughter performance of goslings (%)^1^.

	Dietary paddy rice						
Item^2^	0	13	26	39	52	SEM	*P-*value
Carcass yield	88.60	90.22	88.99	88.55	88.10	0.396	0.536
Semi-eviscerated carcass yield	79.29	80.64	79.33	78.93	79.42	0.393	0.728
Eviscerated carcass yield	73.47	74.51	73.21	72.34	72.72	0.332	0.302
Breast muscle yield	6.69	7.07	7.03	6.76	7.07	0.130	0.830
Leg muscle yield	9.69	10.11	9.73	9.59	9.91	0.131	0.763
Abdominal fat yield	2.15^a^	2.77^b^	2.90^b^	2.84^b^	3.01^b^	0.081	0.002

a,b Means with different superscripts within the same row indicate statistically significant difference (*P* < 0.05).

1 Each value represents the mean of 6 replicate pens.

2 Calculated as a percentage of live body weight before slaughter.

The effect of dietary paddy rice on the proximate composition of the breast and thigh muscle of geese at 70 d is shown in Table 5. There were no effects on the moisture, protein, and fat contents of breast and thigh muscles among the 5 treatments (*P* > 0.05).

**Table 5:** Effects of paddy rice on meat proximate composition of goslings (%)^1^.

	Dietary paddy rice						
Item	0	13	26	39	52	SEM	*P-*value
Breast muscle							
Moisture	76.71	76.07	75.32	75.92	75.94	0.244	0.541
Crude protein	19.81	19.80	21.25	20.68	20.45	0.240	0.257
Crude fat	3.18	4.68	3.88	3.44	3.68	0.383	0.795
Thigh muscle							
Moisture	73.89	73.83	72.99	74.97	73.84	0.668	0.938
Crude protein	22.17	23.17	22.78	22.86	23.24	0.181	0.348
Crude fat	1.40	1.79	2.32	1.34	1.79	0.141	0.181

^a,b^Means with different superscripts within the same row indicate statistically significant difference (*P* < 0.05).

1 Each value represents the mean of 6 replicate pens.

### Meat Quality

The meat quality of the breast and thigh muscles of the geese is presented in Table 6. Compared to the control group, the breast muscle of geese fed the paddy rice groups had a lower L* value and a higher a* value (*P* < 0.05). However, the b* value, pH value, cooking loss, and shear force were not affected by dietary paddy rice (*P* > 0.05). There were no effects on the meat quality of thigh muscle among 5 treatments (*P* > 0.05).

**Table 6:** Effects of paddy rice on meat quality of goslings^1^.

	Dietary paddy rice						
Item	0	13	26	39	52	SEM	*P-*value
Breast muscle							
L*	47.92^b^	43.72^a^	42.60^a^	43.83^a^	42.19^a^	1.071	0.008
a*	11.63^a^	13.28^b^	12.86^b^	13.18^b^	13.72^b^	0.327	0.003
b*	4.22	3.96	3.49	3.83	3.95	0.325	0.648
pH value	6.56	6.20	6.15	6.25	6.21	0.121	0.244
Cooking loss, %	35.52	35.53	33.35	31.75	34.82	0.939	0.116
Shear force, N	126.07	128.87	126.74	119.62	128.4	8.341	0.938
Thigh muscle							
L*	43.90	42.90	42.52	43.03	42.23	0.821	0.668
a*	12.36	13.12	12.64	12.96	13.79	0.529	0.415
b*	4.72	4.68	4.64	4.64	5.16	0.353	0.822
pH value	6.28	5.99	6.11	6.20	6.07	0.037	0.117
Cooking loss, %	32.09	31.91	33.35	34.87	32.71	1.282	0.314
Shear force, N	63.94	48.54	65.93	43.00	53.61	7.148	0.148

a,b Means with different superscripts within the same row indicate statistically significant difference (*P* < 0.05).

1 Each value represents the mean of 6 replicate pens.

### Bare Skin Color

**Figure 1:**
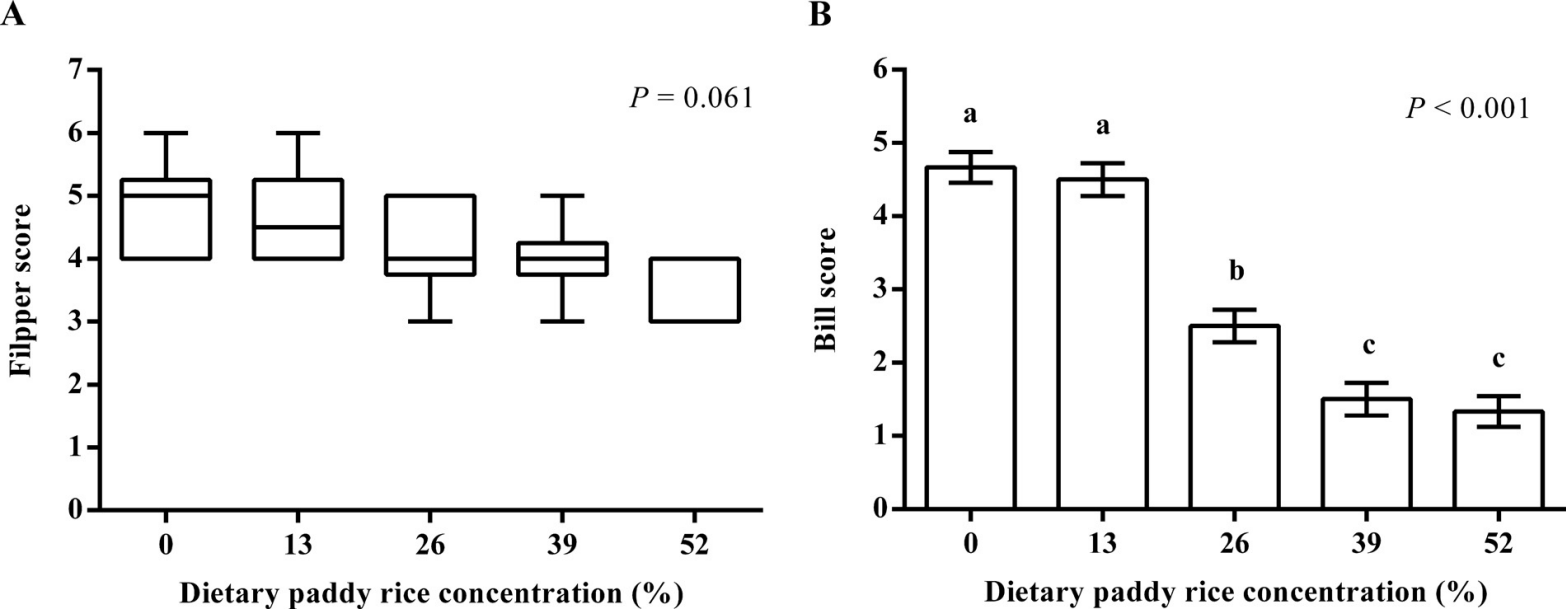
Effects of paddy rice on flipper and bill score of goslings (n = 6). The higher the color score is, the more yellow the bare skin color; the lower the score is, the paler the color. (A) Statistical testing was carried out with Kruskal-Wallis test and the results are presented as box plots (median, first, and third quartiles). (B) Statistical testing was carried out with one-way ANOVA followed by Duncan's multiple-range test and the results are presented as the means and SEM. Different letters on bars indicate statistically significant difference (*P* < 0.05).

A Kruskal-Wallis test showed that dietary paddy rice did not significantly affect the bare skin score of the flipper (*P* > 0.05; Figure 1A). One-way ANOVA showed that dietary paddy rice decreased the bill score (*P* < 0.05; Figure 1B). The control and 13% dietary paddy rice groups had a higher bill score than 26, 39, and 52% dietary paddy rice groups (*P* < 0.05); and the 26% dietary paddy rice group had a higher bill score than 39 and 52% dietary paddy rice groups (*P* < 0.05). The higher the color score is, the more yellow the bare skin color; the lower the score is, the paler the color.

### Nutrient Digestibility

As shown in Table 7, the geese in the paddy rice groups exhibited significantly higher digestibility of total starch than the geese in the control group (*P* < 0.05). However, the utilization of other nutrients, including energy, crude protein, crude fat, crude fiber, NDF, and ADF, did not differ between groups (*P* > 0.05).

**Table 7:** Effects of paddy rice on nutrient digestibility of goslings (%)^1^.

	Dietary paddy rice						
Item	0	13	26	39	52	SEM	*P-*value
Energy	80.81	81.41	81.50	80.31	79.63	0.414	0.602
Dry matter	76.49	76.46	76.34	74.79	73.82	1.791	0.508
Crude protein	78.36	80.82	80.09	80.04	77.59	0.790	0.702
Crude fat	84.93	83.07	76.80	79.85	76.20	1.616	0.367
Crude fiber	16.41	14.32	18.51	17.84	16.95	1.119	0.835
Neutral detergent fiber	37.28	31.57	39.58	32.60	30.02	1.468	0.221
Acid detergent fiber	17.36	16.61	20.73	19.67	21.27	1.018	0.544
Total starch	92.08^a^	94.29^b^	94.11^b^	93.88^b^	94.54^b^	0.276	0.017

a,b Means with different superscripts within the same row indicate statistically significant difference (*P* < 0.05).

1 Each value represents the mean of 6 replicate pens.

## DISCUSSION

To alleviate the increasing global demand for corn, many types of grains are being used to replace corn as a source of energy in feed. For poultry, paddy rice is an excellent energy source among many grains. Sittiya and Yamauchi (2014) found that whole-grain paddy rice can be diluted by up to 40% as a feed ingredient in chicken basal diets. In the 2 trials of Sittiya et al. (2014, 2015), whole-grain paddy rice could replace corn up to a level of 50%, and whole-grain paddy rice and whole-grain brown rice totally replaced corn in broiler diets without negatively affecting growth performance. In the present study, the growth performance of goslings was significantly influenced by dietary paddy rice inclusion. The goslings fed a diet containing 26, 39, and 52% paddy rice increased the final BW, ADFI, and ADG from 28 to 70 d. This observation is similar with that of Mateos et al. (2007), who reported that the substitution of corn for rice improved ADFI and ADG of the piglets but did not affect F/G. These results indicate that paddy rice can improve the growth performance of goslings by increasing feed intake. However, Zhang et al. (2021) reported that the use of paddy rice did not promote the growth performance of goslings aged 1 to 28 d. One possible reason for these divergent results is the difference in the age of the geese used. The digestive tract of younger goslings is not fully developed, which affects their digestion and utilization of paddy rice.

Slaughter performance is an important index to evaluate the meat production capacity of livestock and poultry, which can directly reflect the differences in nutrient deposition in different parts and tissues of animals. In the present study, carcass yield, semi-eviscerated yield, eviscerated yield, breast muscle yield and leg muscle yield of the geese did not change with increasing dietary paddy rice, indicating that deposition of material in these parts was synchronized with the increase in BW. In this study, however, abdominal fat yield in dietary paddy rice groups was higher than that of the control group. The reason may be related to the difference in proportions of starch fractions and starch granule size in the diet. Rice has more rapidly digestible starch and less slowly digestible starch and resistant starch than corn (Giuberti et al., 2012; Doti et al., 2014). Moreover, rice has faster starch digestion and greater starch digestibility than corn (Tester et al., 2004; Svihus et al., 2005). This was confirmed by starch digestion trials of common cereals in vitro (Giuberti et al., 2012; Doti et al., 2014). In this study, the utilization of total starch in the paddy rice group was higher than that in the control group. The high digestibility of starch determines the level of blood glucose in the first 2 h after feeding that is associated with the insulin response, which in turn leads to more nutrients being allocated to fat deposition (Roberts, 2000; Bolhuis et al., 2008). More feed intake and more nutrients allocated to fat deposition led to an increase in the abdominal fat yield in geese. Li et al. (2019) also reported that the abdominal fat weight and deposition rates of broilers fed wheat-based and rice-based diets were higher than those of broilers fed corn-based diets, although these differences were not significant. Similarly, Doti et al. (2014) confirmed that carcasses of pigs fed barley/broken rice diet had a wider backfat depth than those of pigs fed a barley/corn or barley/peas diet.

However, increased abdominal fat was not related to a higher intramuscular fat content (Doti et al., 2014). This suggests that changing the source of starch is not an effective way to change muscle fat. In the current study, the geese fed a paddy rice diet had similar muscle proximate composition compared with the control group. Similar results were reported for growing pigs by Doti et al. (2014), who noted that dietary starch sources had no effect on moisture, protein and intramuscular fat content in the *longissimus thoracis* muscle of pigs.

Meat quality is closely related to the marketing of meat as well as its processing. For consumers, color and overall appearance are the initial criteria affecting preference when purchasing raw meat products. Higher a* values and lower L* and b* scores mean less pale meat and better quality (Abouelezz et al., 2019). In the current study, goose breast muscle in the paddy rice group exhibited higher a* values and lower L* values, indicating that paddy rice improved breast muscle color. However, the meat quality of thigh muscle was not altered by increasing proportions of paddy rice. The thigh muscle of geese might not be as sensitive as the breast muscle to changes in dietary composition.

People in some regions of China prefer to buy live birds and therefore pay more attention to the appearance of the goose, especially the color of the flipper and bill. In this study, the replacement of dietary corn with paddy rice resulted in a lighter yellow coloration of the bill of goslings. Similar results were reported for broken rice by Chen et al. (2020). The color change may be related to the yellow pigment in corn (Nanto et al., 2012). The yellow pigment of corn is due to carotenoids, mainly composed of polyoxy, zeaxanthin, lutein, cryptoxanthin, zeinoxanthin, and carotenes (Grogan and Blessin, 1968). Poultry cannot synthesize pigments, such as xanthophylls or carotenoids, by themselves but can only obtain them from their feed. Therefore, when using paddy rice as feed, it is important to pay attention to changes in color, as changes in skin color can affect consumer purchase intention.

In summary, goslings fed a diet containing 26, 39, and 52% paddy rice had a higher final BW, average daily feed intake, and average daily gain than those in the control group. Paddy rice improved the breast muscle color and the utilization of total starch but increased the abdominal fat yield and decreased the bill color of geese.
